# Occupational Health: Will Work for Air

**Published:** 2006-06

**Authors:** John Manuel

Indoor air specialists Olli Seppänen of the Helsinki University of Technology and William Fisk and Q.H. Lei of the Lawrence Berkeley National Laboratory are not the first to establish a link between work performance and ventilation—for several decades, researchers have seen an association between an inadequate supply of outdoor air and discomfort and illness among building occupants. But Seppänen and colleagues, in a meta-analysis published in the February 2006 issue of *Indoor Air*, are the first to present a model showing the quantitative relationship between these two variables. Their findings are simple: if you want your workers to perform, you have to let them breathe fresh air.

Ventilation rates vary considerably within and among commercial buildings due to such factors as equipment design and operation. Experts say these rates are often below levels recommended by groups such as the American Society of Heating, Refrigerating, and Air-Conditioning Engineers (ASHRAE). However, says Charlene Bayer, a principal research scientist at Georgia Tech Research Institute, the results seen by Seppänen and others suggest that even the ASHRAE standard is probably not high enough. Further, with the recent spike in oil and natural gas prices, building managers may be keeping ventilation rates intentionally low to save on energy bills—a practice that robs Peter to save Paul, as worker productivity can end up dropping.

The researchers subjected the data from nine earlier studies to statistical analysis to compare the results across studies. Five studies collected data from call centers, one was conducted in school classrooms, and three were conducted in a controlled simulated office setting. Each study compared performance at a minimum of two different ventilation rates.

From each study, Seppänen and colleagues calculated a “performance change” parameter by subtracting performance at the lower ventilation rate (expressed in liters of air per second [L/s]) from performance at the higher ventilation rate and dividing the difference by performance at the lower ventilation rate. (The performance figures were expressed in terms of speed of work, and the change in speed was expressed as a percentage.) The resulting parameter was further normalized by dividing by the difference between the two ventilation rates and multiplying by 10.

Results typically showed increases in average work performance in the range of 1–3% for each 10 L/s-per-person increase in outdoor ventilation rate. The performance increase was greater when ventilation rates were initially low (below 20 L/s per person, which is twice the ASHRAE standard) and almost negligible when ventilation rates were already high (above 45 L/s per person). The authors speculate that the improvement of performance was related to reducing levels of indoor air pollutants.

Will this analysis encourage those who design and manage office buildings to let more outside air flow to their occupants? Not in the short run, says Bayer. “Concerns are still primarily with energy conservation, and are increasing due to continually increasing energy costs.” But, she says, this new analysis provides those who are interested with a tool to better balance the needs of energy conservation and worker health and performance.

## Figures and Tables

**Figure:**
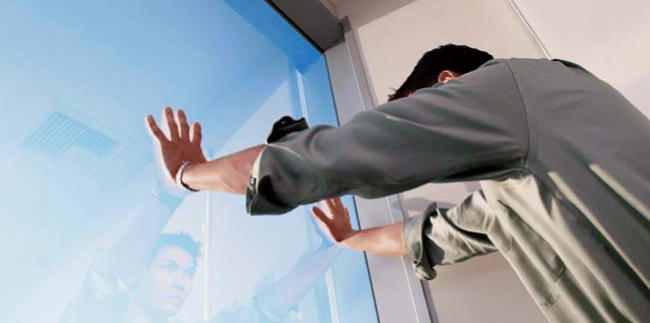
No air, no work Reducing ventilation to save money ends up costing more in lost productivity.

